# Pregnancy‐Associated Acquired Hemophilia A (PA‐AHA) Treated Successfully With the Factor‐Mimetic Bispecific Antibody Emicizumab: A Case Report and Literature Review

**DOI:** 10.1155/crh/7527831

**Published:** 2026-10-08

**Authors:** E. Mandala, A. Barmpouti, K. Lafara, K. Tsioni, T. Achladas, K. Kyrka, E. Gavriilaki, C. Lafaras, S. Vakalopoulou

**Affiliations:** ^1^ Forth Department of Medicine Faculty of Medicine Hippokration Hospital Aristotle University of Thessaloniki Thessaloniki Greece auth.gr; ^2^ Second Propaedeutic Department of Medicine Faculty of Medicine Hemophilia Center Hippokration Hospital Aristotle University of Thessaloniki Thessaloniki Greece auth.gr; ^3^ Theagenio Cancer Hospital Thessaloniki Greece theagenio.gov.gr

**Keywords:** acquired hemophilia A, donor oocytes, emicizumab, immunosuppressive therapy (IST), in vitro fertilization (IVF), pregnancy

## Abstract

Acquired hemophilia A (AHA) is a rare bleeding disorder characterized by spontaneous bleeding in individuals with no previous personal or family history of bleeding, caused by the development of autoantibodies against coagulation Factor VIII (FVIII). These autoantibodies may be associated with drugs, autoimmune disorders, malignancies, and pregnancy. Pregnancy‐associated AHA (PA‐AHA) is a very rare condition, accounting for approximately 1%–5% of all AHA cases. Emicizumab, a bispecific FVIII‐mimetic monoclonal antibody, was originally approved for Congenital Hemophilia A (with or without FVIII inhibitors) and has since gained widespread recognition and clinical utility in the management of AHA as well. We report a case of PA‐AHA that was successfully managed with emicizumab. A 46‐year‐old woman was admitted with vaginal bleeding and extensive ecchymoses. The patient’s obstetric history was notable for a twin gestation achieved through in vitro fertilization (IVF) using donor oocytes. The patient reported recurrent ecchymoses and menorrhagia beginning in the postpartum period. Laboratory examination revealed severe anemia with normal platelet counts and prothrombin time (PT), but a markedly prolonged activated partial thromboplastin time (aPTT) of 94.5 s. Immunological and virology testing, as well as cancer biomarkers, were negative. Initially, the patient received bypassing hemostatic therapy with recombinant activated Factor VII (rFVIIa), along with intensive immunosuppressive therapy (IST) consisting of corticosteroids, rituximab, and cyclophosphamide, in accordance with current international recommendations. However, no clinical response was observed. Treatment with emicizumab was subsequently initiated according to the proposed dosing schedule. Within 2 days, all bleeding manifestations resolved rapidly. Notably, FVIII activity, measured using a chromogenic assay, remained markedly reduced. After 566 days of follow‐up, FVIII activity spontaneously normalized, allowing discontinuation of emicizumab. No further bleeding episodes were observed. Although emicizumab is increasingly incorporated into the management of AHA, clinical experience in PA‐AHA remains limited. The present case adds to the existing literature by supporting the safety and efficacy of emicizumab in PA‐AHA, highlighting its role as an effective hemostatic bridge to remission in complex clinical settings.

## 1. Introduction

Acquired hemophilia A (AHA) is a rare autoimmune bleeding disorder caused by the development of neutralizing autoantibodies against coagulation factor VIII (FVIII). The annual incidence is estimated at 1.48 cases per million population [1]. Although AHA predominantly affects adults, with a median age at diagnosis ranging from 64 to 78 years, it can occur at any age, with two distinct peaks in incidence: one during pregnancy and the postpartum period and another in older adults, particularly those over 60 years of age.

More than 50% of patients with AHA have no identifiable underlying predisposing factors. A clear association has been described with pregnancy and the postpartum period, during which hormonal changes, immune system adaptations, and alterations in hemostatic balance may contribute to disease onset. Autoimmune disorders, including rheumatoid arthritis and systemic lupus erythematosus (SLE), have also been associated with an increased risk of AHA. These conditions are characterized by immune dysregulation and the production of autoantibodies against various proteins, including clotting factors. AHA has additionally been associated with malignancies and certain medications, including penicillin, phenytoin, and interferons [2].

Pregnancy‐associated AHA (PA‐AHA) is particularly rare, accounting for approximately 1%–5% of reported AHA cases. Estimates suggest that PA‐AHA occurs in approximately one in 20.000 to one in 50.000 pregnancies. However, these estimates may vary across populations and geographic regions according to genetic predisposition, underlying medical conditions, and differences in diagnostic criteria and study methodology [3]. PA‐AHA may develop during pregnancy, although it most commonly presents in the postpartum period, typically within 1–4 months after delivery, and may occur as late as 1 year postpartum.

The clinical manifestations of AHA are highly variable, ranging from minimal or absent spontaneous bleeding to severe and potentially life‐threatening hemorrhagic events. The most common bleeding manifestations include subcutaneous hematomas (80%), intramuscular bleeding (45%), gastrointestinal bleeding (21%), genitourinary bleeding (9%), and retroperitoneal bleeding (9%) [4]. PA‐AHA typically presents with unexpected, excessive, or prolonged vaginal bleeding and/or excessive soft‐tissue hematomas, which may be life‐threatening. Prompt recognition and effective management are essential because uncontrolled hemorrhage may result in significant morbidity and mortality.

The standard of care involves the use of hemostatic agents to control acute bleeding, including bypassing FVIII agents such as recombinant activated FVIIa (NovoSeven), activated prothrombin complex concentrate (APCC; FEIBA), or recombinant porcine FVIII (rpFVIII), to manage potentially fatal bleeding, along with immunosuppressive therapy (IST), aimed at eradicating the inhibitor and suppressing anti‐FVIII autoantibody production. IST typically consists of corticosteroids alone or in combination with rituximab or cyclophosphamide. The overall mortality rate of AHA approaches 20% and is associated primarily with severe or uncontrolled bleeding, thrombotic complications related to hemostatic therapy, and, importantly, infectious complications resulting from intensive immunosuppression, which represent a leading cause of death in the majority of registries [5].

Emicizumab is a recombinant, humanized, nonfactor bispecific monoclonal antibody that simultaneously binds activated factor IXa (FIXa) and activated factor *X* (FXa), thereby mimicking the cofactor activity of FVIII and restoring thrombin generation independently of FVIII. It was licensed in 2018 for routine prophylaxis in adult and pediatric patients with hemophilia A, with or without FVIII inhibitors [5]. Recent prospective trials and expert consensus recommendations have further supported the use of emicizumab in AHA. Here, we report a case of PA‐AHA following a twin pregnancy achieved through in vitro fertilization (IVF) with donor oocyte transfer, which was successfully treated with emicizumab.

## 2. Case Report

A 46‐year‐old female was admitted 11 months postpartum with persistent vaginal bleeding and extensive ecchymosis of the left thigh. Eleven months earlier, she had undergone a successful twin pregnancy achieved through IVF with donor oocytes, followed by an uncomplicated cesarean section. Her obstetric history was notable for recurrent miscarriages and multiple failed IVF attempts. Although formal diagnosis was made at 11 months postpartum, the patient reported multiple episodes of spontaneous ecchymoses, menorrhagia, and unexplained fatigue during the preceding months, beginning in the postpartum period, indicating a diagnostic delay prior to her acute hospital presentation. Her medical history was otherwise notable for Hashimoto’s thyroiditis.

Laboratory analysis revealed severe anemia (hemoglobin 6.5 g/dL), with normal platelet counts and prothrombin time (PT), but a markedly prolonged activated partial thromboplastin time (aPTT) of 94.5 seconds. Fibrinogen, D‐dimers, and C‐reactive protein (CRP) levels were elevated, while renal and hepatic function was normal. An initial 1:1 mixing study with normal pooled plasma demonstrated persistent prolongation of the aPTT both immediately after mixing and following a 2‐h incubation period, strongly suggesting the presence of an acquired coagulation inhibitor. AHA was subsequently confirmed by a clot‐based FVIII activity of < 0.25% and a FVIII inhibitor titer of 163 Bethesda units (BU)/mL. Activities of factors IX, XI, and XII, as well as von Willebrand factor (vWF) antigen and activity, were within normal limits.

Testing for lupus anticoagulant, anticardiolipin antibodies (ACAs), and anti‐*β*2‐glycoprotein I antibodies was negative. Immunological testing, including antinuclear antibodies (ANAs), anti–double‐stranded DNA (anti‐dsDNA) antibodies, antiextractable nuclear antigen (anti‐ENAs) antibodies, and anticyclic citrullinated peptide (anti‐CCPs) antibodies, was also negative. Virological testing for cytomegalovirus (CMV), Epstein–Barr virus (EBV), herpes simplex virus (HSV), varicella–zoster virus (VZV), hepatitis B and C viruses, and human immunodeficiency virus (HIV) was negative. In addition, cancer biomarkers were negative. Thyroid function tests were consistent with hyperthyroidism. Ultrasonography of the left thigh revealed a large soft‐tissue hematoma measuring 11.1 cm × 11.1 cm × 16 cm, without evidence of significant pressure effects or compartment syndrome. Abdominal computed tomography (CT) demonstrated bilateral hemorrhagic ovarian cysts.

Following the diagnosis of PA‐AHA, the patient was promptly treated with the bypassing agent rFVIIa (NovoSeven) at a dose of 70–90 mcg/kg every 2–4 h until effective hemostasis was achieved. In parallel, treatment for hyperthyroidism was initiated with propylthiouracil. Intensive IST was subsequently initiated in accordance with current international recommendations [1]. Given the markedly reduced FVIII activity (< 1%), a dual IST regimen consisting of corticosteroids (prednisolone 1 mg/kg) and cyclophosphamide (1.5 mg/kg/day for 6 weeks) was initiated. Despite treatment, at Week 4, the patient developed a new episode of major bleeding characterized by severe menorrhagia and recurrence of the left thigh hematoma, necessitating blood transfusions. As FVIII remained < 1%, rituximab was added at a dose of 375 mg/m^2^ once weekly for four consecutive weeks. rFVIIa was administered as required to achieve prompt hemostatic control. Chlormadinone acetate combined with ethinylestradiol was also administered to control excessive menstrual bleeding.

Despite these interventions, FVIII activity remained < 2%, while the FVIII inhibitor titer remained elevated at 47 BU/mL, and new ecchymoses were observed. Consequently, mycophenolate mofetil (MMF) was introduced, with gradual tapering of corticosteroid therapy. During this period, the patient developed febrile neutropenia, which was treated with broad‐spectrum antibiotics and granulocyte colony‐stimulating factor (G‐CSF). This was accompanied by the development of new ecchymoses, recurrent vaginal bleeding, and another large hematoma, necessitating further blood transfusions. Given the ongoing risk of bleeding and the anticipated need for prolonged treatment before achieving a response to IST, an off‐label regimen of subcutaneous emicizumab was initiated with the aim of preventing further bleeding episodes and facilitating continuous management in the outpatient setting. Emicizumab was administered at a loading dose of 3 mg/kg once weekly for the first 4 weeks, followed by a maintenance dose of 1.5 mg/kg every two to 4 weeks until remission.

Within 2 days of initiating emicizumab, the aPTT normalized to 28.4 s. This finding reflected the capacity of emicizumab to bypass the intrinsic coagulation pathway and interfere with aPTT‐based one‐stage clotting assays, resulting in an artificially shortened clotting time. All bleeding manifestations also resolved following initiation of emicizumab. One month later, FVIII activity measured using a chromogenic assay unaffected by emicizumab remained undetectable (< 1%). IST with MMF was continued for 1 year, but was ultimately discontinued because of the absence of a measurable response. Despite persistent severe FVIII deficiency, the patient remained clinically stable and led a normal life without further bleeding episodes. Serial monthly assessment using a chromogenic FVIII assay revealed persistently severe FVIII deficiency, with FVIII levels consistently remaining below 5%.

Remarkably, after 566 days, FVIII activity spontaneously returned to the normal range, reaching 73% and 86% on two separate assessments performed 3 weeks apart. In parallel, the FVIII inhibitor became undetectable. Emicizumab was subsequently discontinued. The patient was then closely monitored with complete blood counts, aPTT, and FVIII activity every 2 weeks during the first 3 months, followed by monthly assessments for the subsequent 6 months. At last follow‐up, 3 years post–PA‐AHA diagnosis, she has successfully maintained her health condition and leads a fulfilling, normal life, with no recurrence of bleeding or evidence of inhibitor relapse. A comprehensive timeline summarizing the patient’s clinical evolution, from postpartum disease onset and diagnosis to therapeutic interventions, bleeding complications, and eventual restoration of FVIII activity, is provided in Figure 1.

**FIGURE 1:**
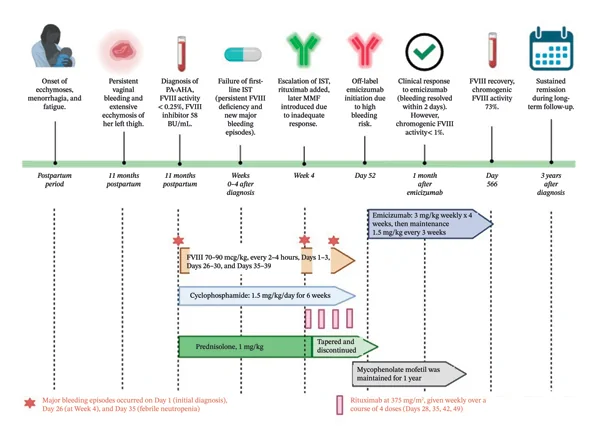
Timeline figure summarizing postpartum onset, diagnosis, IST escalation, emicizumab initiation, bleeding episodes, and FVIII recovery. IST: immunosuppressive therapy; MMF: mycophenolate mofetil; PA‐AHA: pregnancy‐associated acquired hemophilia A; rFVIII: recombinant Factor VIII. This timeline figure was drawn by Thomas Achladas using BioRender (Version 2023, BioRender, Toronto, Canada; https://www.biorender.com/).

## 3. Discussion

AHA is a rare autoimmune bleeding disorder that can cause severe and potentially life‐threatening hemorrhagic events. Although it predominantly affects older adults, a distinct incidence peak has been described during pregnancy and the postpartum period. More than 64% of AHA cases diagnosed in women of reproductive age are pregnancy‐related [6]. The precise mechanisms underlying the development of neutralizing autoantibodies against FVIII remain unclear.

The pathogenesis of FVIII autoantibody formation during pregnancy has not been fully elucidated. One proposed mechanism is that fetal FVIII may act as an immunogenic trigger, initiating an immune recognition cascade and subsequent production of maternal anti‐FVIII antibodies. The higher incidence of PA‐AHA during the postpartum period may be related to maternal exposure to fetal antigens during delivery, potentially promoting the development of FVIII autoantibodies. However, this hypothesis does not fully explain the absence of a clear anamnestic response during subsequent pregnancies, given the low incidence of AHA in subsequent pregnancies [7]. According to international registries and guidelines, PA‐AHA is defined as AHA diagnosed within 12 months of delivery. Notably, a considerable proportion of PA‐AHA cases experience a diagnostic delay, where bleeding symptoms begin during the early puerperium but remain unrecognized until severe hemorrhagic complications occur later in the first postpartum year, as illustrated in our patient [3, 4].

We report an unusual case of AHA diagnosed in the late postpartum period following a twin, dizygotic pregnancy achieved through IVF using donor oocytes. To the best of our knowledge, the only other pertinent case reported in the literature describes AHA occurring in a neonate born to a woman who had conceived following donor oocyte IVF [8]. Pregnancies achieved using donor oocytes are associated with an increased risk of perinatal complications, such as preterm labor, gestational hypertension, and preeclampsia, potentially reflecting the immunological challenges associated with the allogeneic nature of the fetus. Because the fetus may be immunologically more distinct from the mother than in spontaneously conceived pregnancies, donor oocyte pregnancies may involve greater maternal–fetal immune incompatibility and enhanced immune activation. This immunological milieu could potentially contribute to the development of autoimmune complications, including AHA [8–11].

In twin pregnancies, particularly dizygotic pregnancies, as in our case, the presence of two genetically distinct fetuses may further increase the degree of fetal antigen exposure and maternal–fetal immunological complexity. Nevertheless, it must be acknowledged that a direct link between donor oocyte pregnancy and maternal FVIII autoantibody generation remains a speculative hypothesis, as robust evidence supporting a causal relationship is currently lacking. In our case, preexisting Hashimoto’s thyroiditis constitutes a well‐established primary predisposition to autoimmune disruption and may therefore provide a more plausible explanation for the development of AHA. We hypothesize that the combination of thyroid autoimmunity, acting as a principal underlying autoimmune predisposition factor, along with the potential immunological trigger associated with a donor oocyte twin pregnancy, may have contributed to the development of AHA.

IST is recommended for all patients with AHA, according to current international recommendations [1]. Recent studies have demonstrated higher complete remission (CR) rates with combination therapy compared with corticosteroid monotherapy. Specifically, CR rates of approximately 35.2% have been reported with corticosteroid monotherapy, compared with 67.7%–83.3% with combination regimens [12]. Accordingly, current international AHA recommendations support the addition of either rituximab or cyclophosphamide to corticosteroid‐based therapy as first‐line treatment in patients with FVIII activity < 1% [1].

Approximately 70% of patients with AHA require hemostatic treatment. In the EACH2 Registry, the response to hemostatic therapy among 42 women with PA‐AHA was comparable to that observed in patients with AHA of other etiologies [6]. Approximately 70% of these women received corticosteroids alone, while the remainder received corticosteroids in combination with rituximab, cytotoxic agents, or intravenous immunoglobulins. Rates of remission and time to remission were comparable between women with PA‐AHA and the broader AHA population. Nevertheless, overall survival was significantly better among women with PA‐AHA than in the overall EACH2 cohort (survival 100% vs. 78%, respectively, *p* < 0.001) [6]. This favorable outcome may be attributable, at least in part, to the younger age of patients with PA‐AHA and the transient nature of the pregnancy‐associated trigger. The risk of relapse also appears to be relatively low. Therefore, there is currently no evidence to support a fundamentally different IST strategy specifically for PA‐AHA.

Delayed responses to IST appear to be relatively common in PA‐AHA. The median time to first response has been reported as 3 months (range, 0–131 months), whereas the median time to optimal response is approximately 7 months (range, 1–37 months) [12, 13]. In the EACH2 Registry, the time to CR following first‐line IST did not differ significantly between patients with PA‐AHA and the overall AHA population. Nevertheless, other studies have reported a relatively delayed response in PA‐AHA, with a median time to first response of approximately 3 months and to optimal response of 7 months, compared with a median time to partial remission of approximately 5 weeks (range, 1–52 weeks) in broader AHA populations [14, 15].

Interestingly, in several cases, spontaneous remission of PA‐AHA has also been reported, occurring at a median of approximately 30 months in some cases [7]. These observations highlight the complex and potentially self‐limited nature of PA‐AHA. The transient nature of the underlying trigger, namely pregnancy, may contribute to the favorable long‐term outcomes observed in this population. Our patient had a particularly protracted disease course, with spontaneous recovery of FVIII activity occurring after 566 days. Although the precise mechanism underlying this delayed remission remains unclear, the immunological consequences of the preceding twin pregnancy may have contributed. Fetal cells are known to persist in maternal tissues for years after delivery, a phenomenon referred to as fetal microchimerism. In pregnancies achieved using donor oocytes, the presence of genetically distinct fetal cells may introduce additional complexity and potentially influence long‐term maternal immune responses [8, 9]. Nevertheless, the association between fetal microchimerism, donor oocyte pregnancy, and the development or resolution of AHA remains speculative and requires further investigation.

Emicizumab is a first‐in‐class FVIII‐mimetic bispecific monoclonal antibody that bridges FIXa and FXa, thereby mimicking the cofactor activity of FVIII and promoting thrombin generation in the absence of endogenous FVIII. In addition to its impressive efficacy in preventing bleeding, emicizumab has demonstrated a favorable safety profile in patients with congenital hemophilia A. Its subcutaneous administration and prolonged half‐life make it an attractive option for long‐term prophylaxis [16, 17]. In our patient, emicizumab was well tolerated, with no evidence of treatment‐related adverse events, including thromboembolism or thrombotic microangiopathy.

Several case reports and small patient series have described the off‐label use of emicizumab in AHA over recent years [16]. In a multicenter case series involving 32 medical centers in the United States, 40 of 358 patients with AHA received emicizumab. The median age was 73 years (range, 34–87 years) and 95% of patients tolerated treatment without reported complications. In another case series of 12 patients with AHA treated with emicizumab in combination with low‐intensity immunosuppression, a rapid and substantial reduction in bleeding events was observed, with a median time to bleeding control of 3 days (range, 2–15 days) [17]. Clinical remission was subsequently achieved at a median of 105 days (interquartile range, IQR 9–270 days).

More recently, results from the open‐label, single‐arm, Phase 2 GTH–AHA–EMI clinical trial, conducted across 16 Hemophilia Centers in Germany and Austria, have provided prospective evidence supporting the efficacy of emicizumab in AHA. A total of 47 patients with newly diagnosed AHA received emicizumab without concomitant IST. The primary endpoint was the number of clinically relevant bleeds per patient‐week until Week 12. No clinically relevant bleeding events were observed in 33 (70%), while one bleeding event occurred in seven patients (15%), two events in six patients (13%), and three events in one patient. The primary endpoint of the study was achieved with a mean bleeding rate of 0.04 events per patient‐week through Week 12 [18]. Similarly, the Japanese CTI‐205151 open‐label Phase III study evaluated the efficacy, safety, and pharmacokinetics of emicizumab in AHA. Twelve patients were enrolled, of whom 11 (91.7%) subsequently received IST after completion of emicizumab treatment. No major bleeding events were reported, although one nonserious deep vein thrombosis (DVT) occurred [19].

Based on these findings from the above‐mentioned studies, along with accumulating evidence from individual case reports and patient series, the GTH‐AHA Working Group has recently developed consensus recommendations regarding the use of emicizumab in AHA [20]. These guidelines support emicizumab as a prophylactic strategy for the prevention of bleeding episodes in patients with AHA, while emphasizing that IST should be initiated as soon as the patient’s clinical condition permits [21, 22]. However, several important questions remain unresolved, especially regarding the thrombotic risk associated with emicizumab in AHA patients treated with emicizumab and its potential use during pregnancy and the peripartum period, where clinical experience remains extremely limited. The off‐label use of emicizumab in AHA raises several practical considerations, including the optimal dosing regimen, appropriate timing of treatment initiation and discontinuation, patient selection, the management of breakthrough bleeding, the concomitant use of bypassing agents, and the most appropriate laboratory monitoring strategy [23, 24].

Breakthrough bleeding appears to be uncommon among patients with AHA receiving emicizumab. In our review of 207 patients treated with emicizumab, 26 patients (12.5%) experienced bleeding events of any type during treatment (Table 1). A comprehensive review of the literature describing the use of prophylactic emicizumab in AHA is presented in Table 1 [17–19, 25–47]. To compile the data presented in Table 1, we searched PubMed and Google Scholar for English‐language articles published from 1995 to 2025. About 49 references were reviewed. We included clinical trials, observational studies, and case reports detailing the use of emicizumab and clinical outcomes in adult AHA patients. The keywords used were as follows: acquired hemophilia A, pregnancy, emicizumab, IST, IVF, and donor oocytes.

**TABLE 1:** Our comprehensive review of the literature identified cases of AHA managed with prophylactic emicizumab, PA‐AHA: 6/207 patients.

Reference No	Patient No.	Emicizumab dose	Patient gender and age	Underlying condition	FVIII activity and FVIII inhibitor titer, IU/dL (median and range) at diagnosis	Immunosuppressive regiments	Bypassing agents used	Breakthrough bleeding events after emicizumab	Median follow‐up	Time to complete response (CR) or duration of emicizumab treatment	Deaths	Other AE/SAE	Time to CR
25	12 pts	6 mg/kg on Day 1 and 3 mg/kg on Day 2, followed by 1.5 mg/kg/wk from Day 8 onward	6 males/6 females median age (range): 76 (50–92) yrs	3 pts (25.0%): autoimmune diseases, 5 pts (41.7%): malignancies. 4 pts: not defined	FVIII activity: 1.0 (< 0.8–36.6) FVIII inhibitor: 40.5 (1–149) BU/mL	‐ 12 pts: steroids ‐ 3 pts: cyclophosphamide ‐ 9 pts: cyclosporine	rFVIIa/pd‐FVIIa/FX/aPCC	‐ 27 all bleeds in 5/12 pts (41.7%) ‐ Five treated bleeds: in 2/12 pts (16.7%) ‐ No major bleeds	Pretreatment period: 68.0 (17–168) days On‐treatment period: 44.5 (8–208) days, safety follow‐up period: 116.0 (14–170) days	On‐treatment period: 44.5 (8–208) days	No deaths due to bleeding or infection One death unrelated to emicizumab (worsening of CKD and dialysis)	AEs: in 3 patients, ‐ thrombocytopenia (1 patient), ‐ increased prothrombin fragment 1 + 2 (1 patient), ‐ deep vein thrombosis (1 patient). 6 AEs in 4 patients (33.3%), none related to emicizumab	Disease duration for AHA 17.5 (2–2167) days.
26	12 pts	3 mg/kg/wk for 2 to 3 doses, followed by 1.5 mg/kg every 3 wks	6 males/6 females median age (range): 74 (64–80) yrs	‐ 1 pt: Chronic inflammatory bowel disease ‐ 6 pts: Metabolic syndrome, adiposity, diabetes ‐ 2 pts: active infections ‐ 6 pts: Vascular disease ‐ 3 pts: malignant disease ‐ 4 pts: lung disease ‐ 5 pts: Psychiatric disorder	FVIII activity: < 1%; FVIII inhibitor: 22.3 (3–2000) BU/mL	steroids and/or rituximab	aPCC/ rFVIIa/ FVIII concentrate	no		Median time to complete response: 115 days (range, 18–403 days),	No deaths related to emicizumab One death due to exacerbation of chronic inflammatory bowel disease	79‐year‐old Female pt: minor stroke on day 16 on emicizumab during comedication with repetitive single doses of 90 mg/kg rhFVIIa	Median time to clinical remission was 105 days (9–270 days
27	2 pts	Loading dose 3 mg/kg/wk for 2–3 doses followed by maintenance 1.5 mg/kg every 1–3 wks	1 male, 66 yrs 1 female, 64 yrs	*Patient 1:* gout and hypertension *Patient 2*: Type 2 diabetes, chronic kidney disease, gout, and nonalcoholic steatohepatitis	*Patient 1:* FVIII activity: < 1%; FVIII inhibitor: 430 BU/mL (ref< 0.6 BU/mL) *Patient 2:* FVIII activity: < 1% FVIII inhibitor: 353 BU/mL (ref: < 0.6 BU/mL)	Prednisone 1 mg/kg daily and cyclophosphamide 100 mg daily, continued for 6 weeks, with rituximab 375 mg/m^2^ weekly for four doses	rpFVIII, rFVIIa	no		Both pts discontinue emicizumab after 3 months	no	no	3 months
28	4 pts	3 mg/kg/wk for 4 wks, followed by 1.5 mg/kg/wk	2 males/2 females Mean age: 62.3 yrs (50–78)	Multisite autoimmune disease	FVIII activity: < 1%; FVIII inhibitor: 54.3 BU/mL (3.1–81.6);	‐ 4 pts: steroids ‐ 3 pts: rituximab	rFVIIa	no		CR after 24 and 42 days (*n* = 2) Emicizumab stop after 4 injections (median; range: 3–6; *n* = 3)	no	One female pt (age 80 years), with severe comorbidities, major abdominal surgery): minor thromboembolic stroke after the third emicizumab dose (FVIII:chrom = 10%) in association with concomitant rFVIIa therapy for surgery	24 and 42 days (*n* = 2), not reached at the time in the other two case
29	1 pt	3.0 mg/kg/wk (4 wks) + 1.5 mg/kg/wk	1 Female 21 yrs	Autoimmune hemolytic anemia and immune thrombocytopenia, epidermolysis bullosa acquisita	FVIII activity: < 0.25%; FVIII inhibitor: 40 BU/mL	Dexamethasone and cyclophosphamide, ofatumumab, bortezomib, daratumumab	rFVIIa	Bleeding after dental extraction requiring rFVIIa infusions	ND	Not reached	no	no AE	
30	1 pt	3 mg/kg	1 male, 75 yrs	Chronic kidney disease Stage 3b, arterial hypertension, and insulin‐dependent diabetes mellitus, polyneuropathy and a chronic foot ulcer SARS‐CoV‐2 vaccination (AstraZeneca) 10 days before	FVIII activity: < 1%; FVIII inhibitor: 135 BU/mL	Rituximab 375 mg/m2 weekly (2 doses) and methylprednisolone	rFVIIa	no	no		Death unrelated to emicizumab	Death due to comorbidities (new atrial fibrillation, acute kidney injury, and methicillin‐sensitive *Staphylococcus aureus* sepsis)	
31	1 pt	3 mg/kg/wks for 4 wks, followed by 3 mg/kg every 2 wks, after VTE, 1.5 mg/kg every 2 wks	1 male, 75 yrs	Rheumatoid arthritis atrial fibrillation, diabetes mellitus	FVIII activity: < 1%; FVIII inhibitor: 627 BU/mL	Prednisone 1 mg/kg oral daily	aPCC/rFVIIa/rpFVIII	no	9 months	At publication day, CR was not reached after 9 months of emicizumab	no	DVT after several weeks on emicizumab maintenance FVIII activity levels (< 1%), by bovine chromogenic assay	Not reached
32	1 pt	3 mg/kg/wk for the first 3 wks, followed by 1.5 mg/kg every 2–4 wks	1 male, 75 yrs	Severe COVID‐19 disease, hypertension, hyperlipidemia, infrarenal aortic aneurysm, previous STEMI	FVIII activity: < 1%; FVIII inhibitor: 41,8 BU/mL	Prednisone 100 mg, then dexamethasone 6 mg and rituximab (after repeatedly tested positive for anti‐SARS‐CoV‐2 antibodies)	rFVIIa	No	No	CR: achieved between Days 82 and 110, after 4 doses of emicizumab	No	No	Days 83–110
33	4 pts	3.0 mg/kg/wk for 4 wks followed by 1.5 mg/kg/wk	1 Female: 57 yrs, 1 male: 67 yrs, 1 male: 74 yrs, 1 male: 68 yrs	CVD, diabetes, MGUS, dementia	FVIII activity: < 1%; FVIII inhibitor (mean): 68 BU/mL (10–169);	Rituximab (all pts) + corticosteroids (1 pt) + cyclophosphamide (2 pts)	rpFVIII (all pts) + rFVIIa (1 pt)	1 bleeding event requiring hospitalization	247 days	Median time to complete response: 170 days (range, 87–247 days),	No	No	
34,35	60 pts	Loading dose 18 pts*:* 3.0 mg/kg/wk for 4 wks *4 pts:* 6.0 mg/kg/wk, once then 3.0 mg/kg/wk for 2‐3 doses *1 pt:* single dose 3.0 mg/kg *1 pt:* no loading dose followed by a variation of maintenance doses	median age: 71 ys (range 34–93), 30 females (48.4%)	7 pts: autoimmune disease, 6 pts: cancer, 2 pts: peripartum. 1 pt: mild congenital hemophilia A (developed an autoantibody to FVIII) 11 pts: metabolic syndrome, 11 pts: vascular disease, 3 pts: prior venous thrombosis (none on anticoagulation), 1 pt: alcoholic pancreatitis 1 pt: Alzheimer’s dementia, 3 pts: no comorbidities	Median FVIII (range): < 1–23% Median inhibitor titer 74BU/mL range: 0.8–1100 BU/mL	Glucocorticoids (67%), rituximab (54%), cyclophosphamide (17%), mycophenolate mofetil (13%), daratumumab (4%)	Forty‐nine patients (79%) received acute hemostatic treatment prior to starting emicizumab : VIIa, aPCC, and rpFVIII	1 pt: new ecchymoses after the first loading dose, 2 pts: hematuria resolved with hemostatic agents and in one case with a procedure; 1 pt: severe gastrointestinal bleeding 4 months after starting emicizumab (endoscopy and hemostatic agent needed)	ND	Duration of emicizumab treatment media (range): 13 (1–217) wks	4 pts had died (2 were on emicizumab). No deaths were attributed to emicizumab	1 pt: lower extremity deep vein DVT) while on maintenance emicizumab 3 mg/kg every other week. No history of DVT, but was on apixaban for atrial fibrillation until AHA diagnosis.	4 patients had died, of whom 2 were on emicizumab. No deaths were attributed to emicizumab.
36	11 pts	3 mg/kg/wk for 4 wks, 1 pt: continue with the same dose every 2 weeks for 3 months	Median age, yrs (range): 77 (47–93), 5 females/ 6 females,		FVIII activity 1% (range < 1%–9%) and FVIII inhibitor titer: 33 BU/mL (range 9.2–107.5 BU/mL).	Rituximab (11 pt), Prednisone (3 pt)	rFVIIa (8 pt), vascular embolization (4 pt), Tranexamic acid (pt)	One pt with rebleeding (post‐procedural complication), required blood transfusions and tranexamic acid	13.9 months.	Best responses of CR and PR median (range): 163 (63–267)days	No	2 pts with inhibitor recurrence	
37	2 pts	*Patient 1:* 3 mg/kg/wk for 4 wks then once 6 mg/kg *Patient 2:* 3 mg/kg/wk for 4 wks and then every 2 wks for four doses	1 male, 73‐yrs 1 male, 93‐yrs	Pt 1: gout, essential hypertension and chronic alcohol consumption Pt 2: metastatic prostatic adenocarcinoma, hypertension	*Patient 1:* FVIII activity: < 1%; FVIII inhibitor: 15.2 BU/mL (ref< 0.6 BU/mL) *Patient 2:* FVIII activity: < 1% FVIII inhibitor: 11 BU/mL (ref: < 0.6 BU/mL)	*Patient 1:* Methylprednisolone (1‐mg/kg), rituximab (375 mg/m^2^, weekly, four doses in total and cyclosporine (100 mg per day for 1 month). *Patient 2:* Methylprednisolone and rituximab	rFVIIa	No	*Pt 1:* 75 days *Pt 2:* 4 months	*Patient 1*: 75 days after treatment initiation and 28 days after last emicizumab *Patient 2:* 3 months	No	No	Patient 1: 75 days Patient 2: 3 months
38	1 pt	First dose: 3 mg/kg, second dose: 1.5 mg/kg, 7 days after first dose third and last dose: 1.5 mg/kg, 20 days after first dose	1 male, 83 yrs	Heart failure NYHA III, atrial fibrillation, chronic kidney disease Stage 3, and history of repeated deep vein thrombosis and pulmonary embolism	FVIII activity: < 1% FVIII inhibitor: 97 BU/mL	Steroids, rituximab (4 weekly doses of 375 mg/m^2^ and high‐dose intravenous immunoglobulin)	rpFVIII, rFVIIa PCC, FXIII concentrate, fibrinogen	No	ND	20 days	Death, 36 days after hospital discharge and last emicizumab dosing	No	
39	1 pt	Emicizumab 3 mg/kg/wk for 1 month, then to 1.5 mg/kg/wk	Female, 87 yrs	Atrial fibrillation, recent gastrointestinal bleeding	FVIII activity: < 1% FVIII inhibitor: > 100 BU/mL	No information	aPCC	No	2 months	ND	No	No	
40	3 pts	Biweekly injection of emicizumab	3 Females	Postpartum, recent BNT162b2 (Pfizer) vaccination in all patients	ND	2 patients: steroids 1 patient: steroids and azathioprine	rpFVIII/other bypassing agents not specified	no	10 months	< 10 months	No	No	
41	2 pts	*Female patient:* 3.0 mg/kg/wk for 4 wks and then 1.5 mg/kg/wk *Male patent:* 1.5 mg/kg/wk (two doses) then 1.5 mg/kg/wk every 21 days	1 Female, 57 yrs, 1 male, 90 yrs	Multiple comorbidities	FVIII inhibitor: Female: 6.67 BU/mL male:150.4 BU/mL	No information	No information	No	ND	ND	No	No	
42	1 pt	3.0 mg/kg/wk for 4 wks and then 1.5 mg/kg/wk	Middle‐aged woman	HIV	FVIII inhibitor: 50 BU/mL	Corticosteroids, cyclophosphamide, rituximab, cyclosporine	FEIBA, rFVIIa	No	3 years	NA	No	No	
43	1 pt	3.0 mg/kg/wk for 4 wks and then 1.5 mg/kg/wk	1 male, 72 yrs	History of bullous pemphigoid and coronary artery disease	FVIII activity: < 1% FVIII inhibitor: 409 BU/mL	Corticosteroids, rituximab, cyclophosphamide, cyclosporine, azathioprine, bortezomib, mycophenolate, cladribine, and tacrolimus, iv IgG	aPCC, rpFVIII, FEIBA	No	5 months	NA	No	No	

44	1 pt	3 mg/kg/wk for 4 wks then maintenance dose of 1.5 mg/kg every 2 wks	1 male, 91 yrs	Hypertension, benign prostatic hyperplasia, atrial fibrillation, and mitral valve replacement	FVIII activity < 1% FVIII inhibitor 44 BU/mL	Cyclophosphamide and prednisone	rFVIIa	no	ND	6 months	No	No	6 months
45	1 pt	1.5 mg/kg/wk for four doses, then every 2 wks	1 male, 83 yrs	Atrial fibrillation, drug‐induced thrombocytopenia, and CLL	FVIII activity < 1% FVIII inhibitor 197 BU/mL	Rituximab (375 mg/m2/wk for 4 wks), prednisone (70 mg, daily) and cyclophosphamide (75 mg, daily).	rFVIIa tranexamic acid, aPCC	Major bleed 4 days after initiation of emicizumab, no other bleeds after this time point	ND	Complete response after 295 days, after 21 doses, over 271 days	No	No	
46	5 pts	4 pts: 3–4 mg/kg/wk with no maintenance 1 pt*:* 4 mg/kg/wk with maintenance every 2 weeks	Pt 1: F, 88 yrs, Pt 2: M 61 yrs, Pt 3: F, 56 yrs, Pt 4: M, 69 yrs Pt 5: F 71 yrs	1 female—not defined 1 male—not defined 1 female—not defined 1 male—tonsillar cancer 1 female—breast cancer	FVIII activity/inhibitor Pt 1: 9/14 Pt 2: < 1/155 Pt 3: < 1/155 Pt 4: 1/525 Pt 5: 3/. 31	Corticosteroids, cyclophosphamide, rituximab	rVIIa or aPCC, rpFVIII	4 pts: treated bleeds	Median 5 months (2–30)	106 days (89–196) in 2 pts; not achieved	No	No	124 days
47	7 pts	3 mg/kg/wk for 4 weeks, and thereafter every 2 weeks	Median age, yrs (range): 75 (68–78), 5 males/2 females,	Hypertension—5, diabetes mellitus—3, hypercholesterolemia—4, smoking—4, history of cancer—1, autoimmune disease—1	FVIII activity: 0% (IQR 0–1) FVIII inhibitor: 182 BU/mL (IQR 104–228) BU/mL	Rituximab, methylprednisolone	Activated FVIIa	No	ND	All patients recovered with at least 20% FVIIIb after 188 days	1 pt died from sepsis after rituximab and emicizumab	No emicizumab‐related	128 days (IQR 88–173)
17	20 pts	3 mg/kg/wk for 4 weeks, and thereafter every 2–4 wks	11 male, 9 female median age 79 yrs (range 51–87)	ND	FVIII activity: < 1% a FVIII inhibitor: 69 BU/mL (3–2300).	Steroids and rituximab	rFVIIa	No	ND	115 days	2 deaths emicizumab unrelated	No	115 days (median)
48	7 pts	6 mg/kg on Day 1 and 3 mg/kg on Day 2 (loading dose) followed by maintenance dose 1.5 mg/kg weekly from Day 8 onward	Median age, 76 yrs (range 60–87 yrs): 3 males/ 4 females,	Autoimmune diseases—1, hypertension—2, diabetes mellitus—2, dyslipidemia—2, vascular diseases—3	FVIII activity %, median (range):%: 1.0 (< 1–10) FVIII inhibitor, median (range): BU/mL: 622 (1.0–4085)	Corticosteroids, rituximab, cyclosporine	rFVIIa and activated prothrombin complex concentrate	All bleeds: 6/4 pts. Major bleed: 1 pt Subcutaneous hemorrhage—4 Intramuscular hemorrhage—3 Gastrointestinal bleeding—1 Hematuria—1	No	PR, 71% median (range)—days: 43 (8–85); CR, % median (range)—days: 118 (51–172)	No deaths reported	No VTE	
49,18	47 pts	Loading dose: 6 mg/kg (Day 1) and 3 mg/kg (Day 2), followed by a maintenance dose of 1.5 mg/kg per week from Day 8 onward until Week 12	Median age, yrs (range): 76 (66–80), 24 males/ 23 females	Autoimmunity—7, malignancy—6, postpartum period—1, diabetes mellitus—16, renal disorders—13, cardiac disorders—13	FVIII activity, median (IQR), IU/dL: 1.4 (0.3–5.6) FVIII inhibitor, median (IQR), BU/mL: 12.2 (4.1–47.2)	No	rFVIIa and recombinant porcine FVIII	Mean bleeds per patient per week 0.042, Eleven events in 10 emicizumab‐treated patients (21%), no fatal outcome	12 weeks	Clinically relevant new bleeding after 12 weeks were 33% and 67% in emicizumab and IST‐treated patients, respectively (hazard ratio, 0.34; 95% CI, 0.19–0.61; *p* < 0.001)	4 deaths (9%), 1 event attributed to COVID‐19 infection and 2 events attributed to bleeding	Deep vein thrombosis 1 pt and 11 AEs in 10 pts	[53]

*Note:* pd‐FVIIa/FX: plasma‐derived Factor VIIa and *X* mixture, pt: patient, pts: patients, rFVIIa: recombinant activated factor VII, rpFVIII: recombinant porcine FVIII concentrate, wk: week, wks: weeks, and yrs: years. Major bleed: (1) life‐threatening, (2) symptomatic in an important region or major organ (e.g., intracranial, intraspinal, intraocular, retroperitoneal, intraarticular, or pericardiac bleeds, or muscle bleeds associated with compartment syndrome), or (3) associated with a decrease of ≥ 2 g/dL in hemoglobin level or necessitating transfusion of ≥ 2 units of whole blood or packed red cells. Treated bleed: if it was directly followed by the use of a coagulation factor product indicated for “treatment for bleed” without an intervening bleed. Complete response (CR): defined by no clinical bleeding and Factor VIII level > 50 IU/dL, > 50% normal or no Factor VIII inhibitors detected with cessation of IST/resumption at a patient’s usual dose.

Abbreviations: aPCC, activated prothrombin complex concentrate; BU, Bethesda units.

Despite the fact that emicizumab is increasingly incorporated into the therapeutic management of AHA, its clinical application in PA‐AHA remains insufficiently characterized. In our review of 207 AHA cases treated with emicizumab, PA‐AHA cases were rare, accounting for only 2.89% (6/207) of cases. Τhis underscores the limited evidence regarding the clinical application, safety profile, and optimal duration of emicizumab therapy specifically in pregnancy‐related cases, a distinct clinical setting involving younger patients and a disease course that often culminates in spontaneous remission. In conclusion, emicizumab holds significant potential for use in AHA, owing to its distinctive mechanism of action and its favorable safety profile. In this context, our case report adds valuable real‐world data to the existing body of evidence on emicizumab use in AHA and highlights that this regimen can provide complete, long‐term hemostatic protection in a patient with refractory PA‐AHA. Additional safety and dosing data are needed to clarify the role of emicizumab in PA‐AHA.

## Author Contributions

E.M. designed the study. E.M. and A.B. wrote the paper and provided data selection and curation. A.B. performed the literature review and data presentation. T.A., K.L., K.T., and K.K. collected the clinical and laboratory data, performed the literature research, and analyzed the data. T.A. designed the figure and performed editing. E.G. and C.L. performed the critical analysis of the data. E.M. and S.V. provided the final correction of the manuscript.

## Funding

The authors received no financial support for the research, authorship, and/or publication of this article.

## Disclosure

All authors contributed to the correction of the paper, read, and approved the final manuscript.

## Consent

Written informed consent was obtained from the patient for publication of this case report and any accompanying images.

## Conflicts of Interest

The authors declare no conflicts of interest.

## Data Availability

The data underlying this article are available upon reasonable request to the corresponding author.
